# Why patients may not exercise their choice when referred for hospital care. An exploratory study based on interviews with patients

**DOI:** 10.1111/hex.12224

**Published:** 2014-06-17

**Authors:** Aafke Victoor, Diana Delnoij, Roland Friele, Jany Rademakers

**Affiliations:** ^1^ Netherlands Institute for Health Services Research (NIVEL)Utrechtthe Netherlands; ^2^ Quality Institute of the National Health Care InstituteDiementhe Netherlands; ^3^ ‘Transparency in Health Care’Tilburg UniversityTilburgthe Netherlands; ^4^ ‘Social Impact of Health Law’Tilburg UniversityTilburgthe Netherlands

**Keywords:** choice behaviour, hospitals, patient freedom of choice laws, patients, qualitative research, quality of health care

## Abstract

**Background:**

Various north‐western European health‐care systems encourage patients to make an active choice of health‐care provider. This study explores, qualitatively, patients' hospital selection processes and provides insight into the reasons why patients do or do not make active choices.

**Methods:**

Semi‐structured individual interviews were conducted with 142 patients in two departments of three Dutch hospitals. Interviews were recorded, transcribed and analysed in accordance with the grounded theory approach.

**Results:**

Three levels of choice activation were identified – passive, semi‐active and active. The majority of the patients, however, visited the default hospital without having used quality information or considered alternatives. Various factors relating to patient, provider and health‐care system characteristics were identified that influenced patients' level of choice activation. On the whole, the patients interviewed could be classified into five types with regard to how they chose, or ‘ended up at’ a hospital. These types varied from patients who did not have a choice to patients who made an active choice.

**Conclusions:**

A large variation exists in the way patients choose a hospital. However, most patients tend to visit the default without being concerned about choice. Generally, they do not see any reason to choose another hospital. In addition, barriers exist to making choices. The idea of a patient who actively makes a choice originates from neoclassical microeconomic theory. However, policy makers may try in vain to bring principles originating from this theory into health care. Even so, patients do value the opportunity of attending ‘their’ own hospital.

## Introduction

### Background

Patient choice of health‐care provider plays a pivotal role in regulated competition within Western countries' health‐care systems, for instance, to reduce waiting time and introduce competition between providers.^1^, ^2^, ^3^ Patients are viewed as autonomous health‐care consumers^4^ supposedly making active, rational choices between different health‐care providers based on comparative information. Because actively choosing patients prompt providers to compete, patient choice will ultimately lead to more efficient care of higher quality, as policy makers assume.^5^ This line of reasoning originates from the neoclassical microeconomic theory.^6^ Besides the instrumental goal of patient choice, it is also considered a goal in itself. Enabling patients to freely choose a provider allows them to personalize care and is assumed to lead to better patient experiences.^2^, ^7^

A great deal of effort has been put into enabling and stimulating patients to choose their providers.^5^ However, the ability of, and opportunity for patients to make active choices is being questioned. For example, behavioural economic research indicates that actual choosing behaviour generally deviates from the full rationality as assumed by neoclassical microeconomic theories.^8^ Besides, the nature of health care makes it difficult, or unrealistic, for patients to make active choices. The choice of a provider does not concern an isolated, one‐off activity, but is part of the patient's whole care path.^9^, ^10^ Because of these factors, patients tend to visit what is known as the standard or ‘default provider’.^11^ This is often the provider they have most experience with,^12^ the nearest one,^11^, ^13^, ^14^ or the one that their general practitioner (GP) recommended.^3^ The default effect exists also for other domains than health care, such as retirement saving. It means that patients do not make an active choice of a provider but simply visit the standard option without having thought about it deliberately.^8^

Several factors influence whether patients bypass the default provider. These include the degree to which patients play an active role regarding their health care^15^, whether they are aware of the fact that they have the right to choose a provider^16^ and whether they are willing to, or have a reason to, make choices^16^, ^17^, ^18^. Another factor is the availability of alternative hospitals.^19^, ^20^ To exercise choice, patients need alternatives to choose between. The medical specialty patients require constitutes another factor. For instance, patients needing surgical care may be more inclined to make an active choice, because the elective nature of most surgical care allows one to think critically about the options. Chronically ill patients, on the other hand, might prefer the nearest hospital simply because they need continued, local care.^21^ The path of the patient's health care is another factor. For instance, as many patients do not know their diagnosis at the moment of referral and patients' path of health care is a process or cycle of alternating stages of diagnosing, treating and adjusting, there is often no one clear moment at which the choice of a hospital can be made.^9^

### Research focus

We aim to investigate why patients' behaviour does not correspond with the image of the autonomous health care consumer policy makers had in mind when giving patients a key role in promoting competition between health‐care providers. Our research question is ‘How do patients either choose or ‘end up at’ a particular hospital and which factors influence their process of making a choice?’ To answer this question, we held semi‐structured interviews with hospital patients, which enable us to focus on the hospital selection process from the patients' perspective instead of on its outcomes. We can untangle the complexity of patient choice of hospitals, obtain detailed information about the feelings, perceptions and opinions of the patients and get insight into the different processes involved in making a choice. We focus on the choice of a hospital, because quality information and the opportunities to choose are available in this sector. Our study is conducted in the Netherlands. Here, patient choice of providers is encouraged; patients generally live in close proximity to several hospitals; GPs serve as gatekeepers for secondary care; and health‐care insurance covering a patient's hospital costs is mandatory.^22^, ^23^

### What this paper adds

Much research into patient choice assumes that patients do choose a hospital when they need to visit one. Research has investigated, for example, which hospital characteristics patients find important, or focused on parts of the process of making a choice, such as patients' use of information.^11^ Our starting point is a belief that patients differ in their intentions and the opportunities available to them to make a choice. By means of in‐depth interviews with hospital patients, we investigate how patients select or ‘end up at’ a hospital, thus arriving at a classification of patients regarding this subject. The paper enables policy makers to modify their assumptions regarding patient choice. Health care could then be organized in a way that ensures that patients visit the hospital that fits their needs and preferences.

## Method

### Research sample

The method of sampling was purposive.^24^, ^25^ To grasp many different perspectives, the authors collected data from outpatients who attended a variety of Dutch hospitals (Hospital A, B and C) each differing in several factors (Table 1). In each hospital, patients were interviewed from two hospital departments, that is, general surgery and internal medicine. In Hospital A, pancreatic cancer patients were excluded, as it is the only hospital in the locality that can treat these patients. Patient recruitment stopped when data saturation occurred, that is, when no new information on the themes was forthcoming, and we were able to categorize patients into groups based on our understanding of patients' hospital selection process.^24^, ^25^ It was possible, thus, to collect and analyse data concurrently and adapt questions to the themes emerging.

**Table 1:** Hospital characteristics

	Hospital A	Hospital B	Hospital C
Hospital type	Teaching hospital	General hospital	General hospital
Urban/rural	Urban	Rural	Urban
Number of hospital beds	1100	565	584
Number of alternatives ≤10 km around the city where the hospital is located^a^	7	0	2
Neighbouring hospital received negative publicity	Yes	No	No

a Only Dutch hospitals were considered.

### Material

The interviews were semi‐structured. Participants were asked, firstly, about their background characteristics. We asked next an open‐ended question: ‘Why did you visit this specific hospital?’. The interviewer was, with regard to this question, allowed to ask questions in an unscripted manner in order to follow up comments made by patients.^25^ Several topics served as input for further questions: (i) patients' behaviour when choosing, such as whether they searched for information and why, or why not, (ii) which attributes of the provider influenced their choice, for instance, the size of the hospital, (iii) which features of the health‐care system affected their choice, such as the availability of choice, and finally, (iv) which factors relating to the interaction between provider and patient characteristics influenced their choice, for example, patients' health‐care paths.

### Procedure

Patients were invited to participate while seated in the waiting room. They were given a choice of being interviewed in the waiting room or in a separate room. Written consent was obtained prior to the start of each interview. At the end of the interview, the interviewer checked the accuracy of her interpretation with the participant by summarizing the interview.^25^ One of the authors (AV) carried out all the interviews. Participants from the first hospital mentioned were the only ones to receive compensation for completing the interviews (sweets and reimbursement of parking costs). This was because the hospital insisted on this. Participants were not informed in advance about this compensation. All interviews were audio recorded.

### Data analysis

Firstly, the audio recordings were transcribed verbatim (AV) and loaded onto MAXQDA, a qualitative data analysis program.^26^ Secondly, the transcripts were read by AV to gain an overview of emerging patterns in the data.^25^ Thirdly, we developed themes relating to our research question. The development of themes involved open, axial and selective coding, consistent with the grounded theory approach.^24^ The themes and subthemes that emerged concerned patients' levels of choice activation and the factors that determined the extent to which their choice was active. Finally, we classified the patients into five groups that differ in how they chose a hospital or ‘ended up at’ a particular hospital. This classification was based on differences and similarities between patients regarding the levels and the factors that influenced these levels. Two authors (AV and JR) met bi‐weekly to discuss emerging codes.

### Ethical considerations

Our research complied with the Helsinki Declaration. According to the Dutch ‘Medical Research involving human subjects Act’, our study did not require ethical approval from an ethics committee.^27^ Written informed consent was obtained from all interviewees and they were ensured anonymity.

## Results

### Demographics

Interviews were conducted with 142 patients (Table 2). We needed that many interviews, mainly because we aimed at maximum variation to cover the wide range of possible hospital selection processes and wanted to be able to categorize patients into different groups based on these processes. The response rate was 91%. The most important reason for not participating was lack of time. Most patients were aged 40–64, female, native Dutch and had a medium educational level. The mean duration of the interviews was 9.49 min (SD 5.07 min). The duration of the interviews was relatively short, primarily because not much time was needed to acquire all information, but also because patients did not give much thought to the choice of a hospital and therefore found it odd or irritating to talk about the subject extensively. Additionally, some consultations started before the end of the interview.

**Table 2:** Demographic characteristics of the interviewees (*n* = 142)

	Hospital A		Hospital B		Hospital C		
	Surgery (*n* = 19)	Internal medicine (*n* = 26)	Surgery (*n* = 26)	Internal medicine (*n* = 25)	Surgery (*n* = 21)	Internal medicine (*n* = 25)	Total (*n* = 142)
	*n* (%)	*n* (%)	*n* (%)	*n* (%)	*n* (%)	*n* (%)	*n* (%)
Age (years)							
Under 40	9 (47.4)	4 (15.4)	2 (7.7)	4 (16.0)	3 (14.3)	1 (4.0)	23 (16.2)
40–64	8 (42.1)	18 (69.2)	13 (50.0)	13 (52.0)	11 (52.4)	12 (57.1)	75 (52.8)
65–74	1 (5.3)	2 (7.7)	5 (19.2)	1 (4.0)	6 (28.6)	12 (57.1)	27 (19.0)
75 and over	1 (5.3)	1 (3.8)	6 (23.1)	6 (24.0)	1 (4.8)	0 (0.0)	15 (10.6)
Missing	0 (0.0)	1 (3.8)	0 (0.0)	1 (4.0)	0 (0.0)	0 (0.0)	0 (0.0)
Gender							
Male	11 (57.9)	16 (61.5)	10 (38.5)	9 (36.0)	11 (52.4)	9 (36.0)	66 (46.5)
Female	8 (42.1)	10 (38.5)	16 (61,5)	16 (64.0)	10 (47.6)	16 (64.0)	76 (53.5)
Missing	0 (0.0)	0 (0.0)	0 (0.0)	0 (0.0)	0 (0.0)	0 (0.0)	0 (0.0)
Education level							
Low	3 (15.8)	5 (19.2)	8 (30.8)	8 (32.0)	6 (28.6)	4 (16.0)	34 (23.9)
Medium	12 (63.2)	6 (23.1)	9 (34.6)	14 (56.0)	13 (61.9)	8 (32.0)	62 (43.7)
High	4 (21.1)	14 (53.8)	5 (19.2)	3 (12.0)	2 (9.5)	13 (52.0)	41 (28.9)
Missing	0 (0.0)	1 (0.0)	4 (15.4)	0 (0.0)	0 (0.0)	0 (0.0)	5 (3.5)
Ethnicity							
Dutch	9 (47.4)	21 (80.8)	18 (69.2)	19 (76.0)	19 (90.5)	23 (92.0)	109 (76.8)
Western immigrant	3 (15.8)	1 (3.8)	5 (19.2)	6 (24.0)	2 (9.5)	2 (8.0)	19 (13.4)
Non‐Western immigrant	7 (36.8)	4 (15.4)	0 (0.0)	0 (0.0)	0 (0.0)	0 (0.0)	11 (7.7)
Missing	0 (0.0)	0 (0.0)	3 (11.5)	0 (0.0)	0 (0.0)	0 (0.0)	3 (2.1)
Level of activation							
Passive	13 (68.4)	15 (57.7)	17 (65.4)	22 (88.0)	14 (66.7)	19 (76.0)	100 (70.4)
Semi‐active	3 (15.8)	7 (26.9)	5 (19.2)	3 (12.0)	3 (14.3)	2 (8.0)	23 (16.2)
Active	3 (15.8)	3 (11.5)	4 (15.4)	0 (0.0)	4 (19.0)	4 (16.0)	18 (12.7)
Missing	0 (0.0)	1 (3.8)	0 (0.0)	0 (0.0)	0 (0.0)	0 (0.0)	1 (0.7)

Low = primary school or only vocational training; Medium = secondary school or intermediate vocational training; High = tertiary education.

### The level of choice activation

**Figure 1:**
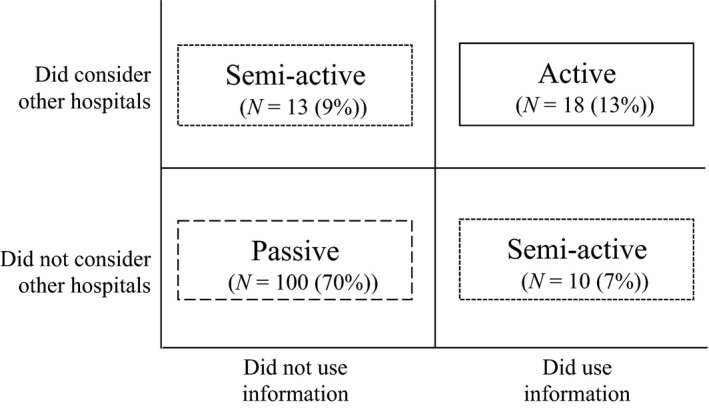
The levels of choice activation.

We found that patients differed in the extent to which their choice was active. Therefore, we used two objective criteria to assess that level. Whether, before visiting a hospital, patients considered other hospitals and, whether they based their choice on a hospital or consultant's reputation or information regarding its or their quality of care (Fig. 1). We identified three levels from the 142 interviews:

1. Passive. Patients did not consider other hospitals before visiting one *nor* based their choice on information regarding the quality of care offered by the hospital or consultant [*n* = 100 (70%)].
2. Semi‐active. These patients considered alternative hospitals *or* based their choice on information regarding the quality of care offered by the hospital or consultant [*n* = 23 (16%)]
3. Active. These patients considered other hospitals before selecting one *and* based their choice on information regarding the quality of care offered by the hospital or consultant [*n* = 18 (13%)].

We did not analyse one interview further as this patient had only come to the hospital because she had participated in a research project. It should be noted that not all patients who made an active choice actually got to visit their preferred hospital. They did not always have the opportunity to choose the hospital of their preference, for instance, because there were not enough hospitals nearby to choose between and there was no space at the preferred hospital [*n* = 7 (5%)].

### Factors influencing the level of choice activation

Various factors led patients to visit the default hospital or stimulated them to make a more active choice. The factors are organized according to the different subthemes that emerged from the interviews:

1. Hospital characteristics: reputation, quality of care and organization.
2. Patient characteristics: attitudes towards making choices.
3. Health‐care system characteristics: information on quality of care and choice options.
4. Factors relating to the interaction between the hospital and the patient characteristics: the opportunity to choose, distance to the hospital and previous experiences.

Illustrative quotes and examples of our findings are provided in the text.

#### Hospital characteristics

##### Reputation

Passive patients indicated that there was no reason to choose, because the default option was good or had a good reputation. These factors led them to be content with the hospital they visited. Therefore, they were not concerned about choosing, actively, a hospital. One patient, for example, indicated that she was not concerned about choosing a hospital, partly because of the good reputation of her current hospital *(woman, 56 years, internal medicine, Hosp.C, passive)*. Alternatively, some patients did not make a choice because of the bad reputation of other hospitals. One patient said: ‘You hear in the news that some hospitals are not good and you know that. But I was already familiar here so I thought, then here’ *(man, 42 years, surgery, Hosp.A, passive)*. Alternatively, patients making active or semi‐active choices mentioned that a bad reputation of the default hospital led them to avoid it.

##### Quality of care

Patients did not see any reason to choose because they either saw no difference in the quality of care offered, thought hospitals will improve to maintain their ranking, or thought that every hospital is able to help them, especially with relatively routine operations. For example: ‘It doesn't really matter to me where I go. They are all good’ *(woman, 62 years, surgery, Hosp.C, passive)*.

Some patients making active or semi‐active choices, however, did think that differences in quality exist, valued their health and were therefore keen to receive the best care. ‘You only have one body, so it should be well taken care of, so I choose the best’ *(woman, 60 years, internal medicine, Hosp.C, active)*. Other patients had bad experiences and so adopted a critical attitude towards hospitals and consultants.

##### Organization

Factors relating to the organization of hospitals also led passive patients to be content with their hospital. Consequently, they were not concerned about choosing, actively, a hospital. For example, their default hospital was a teaching hospital, had all the facilities, had a short waiting time or was small or intimate or located in the Netherlands. One patient explained he visited a hospital because: ‘It is the nearest one for me and, basically, all medical facilities are available here too’ *(man, 73 years, internal medicine, Hosp.C, passive)*.

#### Patient characteristics

##### Attitude towards making choices

Passive patients did not attach much importance to actively choosing a hospital. As one replied to the question whether he thought about choosing a hospital: ‘No, I wanted to be helped as quickly as possible’ *(man, 51 years, internal medicine, Hosp.B, passive)*. This implied that he did not want to waste time on searching a hospital. Patients believed that they could always switch hospitals when dissatisfied; they generally did not like changes; they were easily satisfied; they felt that they were empowered enough to insist upon changes; they did not need to acquire information about quality because they worked as a doctor and; they indicated that there was no common tradition of making a choice. Asked why they did not question the referrer's choice of destination, they answered that they had confidence in their referrer.

#### Health‐care system characteristics

##### Information on quality of care

Passive patients found it difficult to choose. They did not consider themselves experts on the quality of hospitals. One patient declared: ‘This is unknown territory, so then you listen to your GP’ *(woman, 72 years, internal medicine, Hosp.C, passive, 120)*. Patients feared too that searching might uncover incorrect information. However, one patient making an active choice believed it was perfectly possible, because you can find everything on the internet nowadays. She used a search engine to find the hospital that was best able to treat her *(woman, 47 years, internal medicine, Hosp.C, active)*.

##### Choice options

Passive patients also said that there were no alternatives in their locality. This created a problem, especially for patients without a car. One said: ‘Transport was a bit of a problem if I had to travel further and I could still cycle to this hospital or possibly take a bus’ *(woman, 76 years, surgery, Hosp.B, passive)*. A few patients who made an active or semi‐active choice, however, indicated that there are enough hospitals in the neighbourhood to choose between *(man, 24 years, internal medicine, Hosp.B, semi‐active)*.

#### Interaction factors

##### The opportunity to choose

The patients' path of health care made it difficult for them to make a choice. Metaphorically speaking, patients often sat in a moving train and it was hard or illogical for them to get off this train. For instance, some patients did not know their diagnosis in advance or thought that they only had a minor problem. Consequently, they could not choose a hospital that was specialized in their condition or thought that choosing a hospital was unnecessary. Once they were diagnosed, it was easier to stay at their current hospital. As one said: ‘Then he says just go to the hospital, there might be nothing to worry about and then you are already at a particular hospital’ *(woman, 63 years, surgery, Hosp.B, passive)*. Furthermore, patients explained that their current problem was addressed while they were already being treated in a particular hospital or stayed at a particular hospital because they were diagnosed there. Other patients needed to visit a specific hospital, for instance because they needed care urgently and it was the nearest one or it was the only hospital that could treat them.

Other factors also made choosing difficult. Passive patients did not have time to travel to a more distant hospital for the best hospital. They could not make a clear decision once in a doctor's surgery, nor thought critically anymore about mistakes that were made in ‘their’ hospital. Being treated there had now become a habit. Asked if he searched for information about quality, one patient answered: ‘No, working hard, that's what I do’ *(man, 41 years, surgery, Hosp.A, passive)*.

Alternatively, patients making active or semi‐active choices indicated that some situations encouraged choice. These include: when patients need an operation; when they have a severe disease; when they have a specific condition for which specialized care is available and; when their life or health‐care situation changes, such as when they move house. One man said: ‘For serious conditions, I think it is important that it is done well, with the best doctors that are available at that moment’ *(man, 65 years, internal medicine, Hosp.C, active)*. One factor related to the patients' path of health care was that the default hospital could not provide the care they needed. This did not necessarily lead to making a choice, however, as most patients followed their consultant's referral.

##### Distance to the hospital

In addition to hospital characteristics, interaction factors such as the distance to the hospital also caused patients to be unconcerned about choosing, actively, a hospital. For example, ‘I don't have any reason not to go to this hospital. It's the nearest one for us’ *(woman, 63 years, surgery, Hosp.B, passive)*.

##### Previous experiences

Satisfaction or familiarity with the default hospital also caused passive patients to see no reason to make a choice. Factors here include their care history is there; they trust their consultant; they stayed there out of habit; they had bad experiences in other hospitals; they did not have a relationship with another hospital or; they wanted to be hospitalized close to family members. One said: ‘I stay with the same doctor, I find that important. I have faith in that doctor’ *(man, 73 years, internal medicine, Hosp.C, passive)*.

Patients making active or semi‐active choices said that desiring a second opinion and having bad experiences with the default hospital prompted them to bypass this hospital. However, as the following quote illustrates, some patients selected an alternative hospital only for a specific specialty: ‘That trust has been undermined here. So I don't go to the ophthalmologist here anymore’ *(man, 47 years, surgery, Hosp.C, passive)*. Even after a bad experience, some patients stayed with, or went back to, the default hospital once their consultant had apologized.

### Classification of the patients

We classified patients into groups that differ regarding how they chose or ended up at a hospital. This classification was based on differences and similarities between patients regarding their level of choice activation and the factors that influenced the extent to which their choice was active.

1 *Patients with no opportunity to choose because of their health‐care path [n = 20(14%)]*. These patients' care had already begun. Consequently, it felt difficult or illogical to them to make an active choice for another hospital than their current one or the one they were referred to. Others needed to visit a specific hospital, for instance because it was the nearest one and they felt that they needed care urgently. When asked why she visited a particular hospital, one patient answered: ‘There was no choice, I had to go to the nearest one, it was crisis, I had to go to this hospital, done. You don't then look further’. After this first visit, she always visited this hospital for her illness because continuity of care was important for her *(women, 56 years, internal medicine, Hosp.C, passive)*.
2 *Passive patients [n = 41(29%)]*. This type of patient did not attach significance to the choice of a hospital. They did not see any difference in quality between different hospitals nor any reason to switch hospitals. Additionally, they expected their referrer to know best. They automatically visited the hospital they were referred to, which was chosen by a family member, or their nearest hospital, often the only one in the neighbourhood. One patient said: ‘Because my GP referred me to this hospital’ *(woman, 72 years, internal medicine, Hosp.C, passive)*.
3 *Patients choosing the default hospital [n = 42(30%)]*:
  - *Loyal patients [n = 24(17%)]*. Loyal patients always visited the same hospital, the hospital with their care history, or the same hospital as a family member with the same condition. For instance, ‘I find it strange, another hospital’ *(woman, 76 years, internal medicine, Hosp.B, passive)*. It was really ‘their hospital’, they were very satisfied with the care they previously received there and they were convinced that their hospital is good enough to provide the care they needed.
  - *Practical patients [n = 18(13%)]*. These patients focused solely on practical issues. They visited the nearest hospital or the hospital where they work, it being easier therefore to pay a quick visit to the doctor. One said: ‘Because it is the nearest one for me and, basically, all medical facilities are available here too’ *(man, 73 years, internal medicine, Hosp.C, passive)*. Patients were convinced that the hospital they visited was good enough and that there is no reason to think about others.
4 *Patients investing some effort [n = 20(14%)]*. These patients thought about the choice of a hospital or used information about quality, either to confirm the quality of ‘their hospital’ or to select one without having considered other hospitals. One said: ‘They don't have a bad reputation. I did check that’ *(woman, 54 years, internal medicine, Hosp.B, semi‐active)*. Others visited a specific hospital after having considered at least one other hospital, for instance, because it was a teaching hospital.
5 *Patients making active choices [n = 18(13%)]*. These patients were aware of the differences in quality between hospitals and the fact that different hospitals specialize in different fields of care. They attached great importance to their health, thought that it is important to search for the best care, primarily by asking family and friends for their care experiences, and had the opportunity to make an active choice, for instance because they knew their diagnosis in advance. One said: ‘Because of the consultants who work here. They are the best’ *(man, 65 years, internal medicine, Hosp.C, active)*. Some patients, however, were not critical *per se* but were prompted to make an active choice, because, for example, they had a bad experience at the default hospital.

## Discussion

Patient choice of providers is encouraged in various countries in order to stimulate competition between providers, among other goals. However, we found that various factors relating to the characteristics of the patient, the provider, and the health care system influenced patients' hospital selection process. Patients, therefore, select a hospital in a large variety of ways. The patients interviewed could be classified into five patient types, varying from patients who visited the default hospital because they did not have a choice, to patients who correspond to the image of the autonomous health‐care consumer.

### Comparison to the literature

Our finding that the majority of patients visited the default hospital without having used information about quality or considered other hospitals, is consistent with existing literature^11^, ^12^. Many of these patients visited, passively, the default hospital without having thought about the choice of a hospital. Consistent with existing literature,^18^ most of them did not attach significance to the choice of a hospital. For several patients, the choice of a hospital was a trivial issue. They did not see any reason to switch hospitals. They were content with the default hospital or they thought that every hospital should be able to help them. Others did not consider themselves experts on the quality of hospitals. Existing literature also indicates that many patients have trouble with the use of comparative information^28^ and are insufficiently informed to make educated choices.^4^ Some felt that they did not even have the opportunity to make an active choice, for instance, because they did not have alternative hospitals nearby. This is also in line with previous research.^19^, ^20^ Consequently, many patients let their GP or consultant decide on the provider they were to be referred to or simply visited their current or local hospital.^3^ Patients trust their referrer and think that he or she knows best which provider to visit.

Other patients felt that they did not have an opportunity to choose because they had already embarked on a path of care. This is compatible with the ‘logic of caring’, which assumes that the nature of health care makes it difficult or unrealistic to make active choices between health‐care providers.^9^ Neoclassical microeconomic theory assumes that patients have the opportunity to search for the best hospital when a diagnosis is reached. However, according to the logic of caring, patients often do not have one clear moment at which a choice can be made. Even patients visiting the surgery department, who often needed elective care, generally did not make an active choice. This may be explained by the fact that even a specific elective operation does not concern a one‐off, circumscribed event but a process that is part of a patient's life and health‐care path and cannot be separated from previous health‐care experiences.

Thirty per cent of the patients interviewed adopted the default option because of specific reasons. Although these patients regarded the issue of hospital choice as trivial, they still valued the opportunity to visit that particular hospital because they had a relationship with it or because of practical issues. Research has previously shown that patients visit the nearest hospital or the one where they have been before.^3^ Generally, patients are loyal to their local hospital.^14^

Almost three of ten patients were concerned about the choice of a hospital. However, only 13% of the patients interviewed corresponded to the image of the autonomous health‐care consumer. If the default hospital had a bad reputation or patients had a bad experience with it, patients were prompted to switch hospitals. This is consistent with existing literature.^16^, ^17^ Unlike existing literature, however, few patients mentioned negative publicity about a hospital in their locality as a factor that influenced their choice. A few patients, who believed that differences in quality exist between hospitals or that making an active choice is perfectly possible, made an active choice regardless of the situation. Our research indicates that patients only adopt the consumer role in specific situations or with particular characteristics, for instance, if they exhibit a greater capacity for active decision making. Other research shares this conclusion.^15^, ^16^

### Limitations, strengths and further research

Our research is significant because it does not focus solely on patients' preferences regarding hospitals. Instead, it focuses on the assumption that patients differ in their intentions and their opportunities to make a choice. It constructs, from a patients' perspective, a typology of how patients select or ‘end up at’ a particular hospital. Interviewing a large number of patients from two departments of three Dutch hospitals allowed us to grasp many different perspectives.

However, we cannot be sure if our results can be generalized as we did not aim to acquire a sample that is representative of the population of hospital patients. However, our goal was to acquire exploratory in‐depth information rather than to test hypotheses. In our study, we do mention some figures to give an impression about the relative sizes of the different patients groups. These figures need to be interpreted with some caution. Another limitation is that we were unable to draw conclusions about the numbers of patients who made a particular remark. One issue could have been mentioned by all patients or by just a few. However, although a particular issue might have been mentioned by only a few patients, it does not have to be a trivial one. Were we to have asked them about that particular issue, more patients might have considered it an important factor.

This is an exploratory study. Quantitative research should describe the five patient groups as per the various factors that influence the process of making a choice of a hospital and test the differences between the groups regarding those factors.

## Conclusion

Differences exist in the way patients select a hospital even though patient choice of hospitals is encouraged in Western countries. Generally, patients tend to visit the default hospital without having been concerned about the choice of a hospital. This is because they do not see any reason to choose another one. These patients valued choice as a means to be able to visit the default hospital or, in other words, a goal in itself, and not as an instrument to improve care on a macro level. Several barriers to making an active choice were identified. Some barriers might be reduced by taking measures to encourage choice, but other barriers are inherent in the health‐care sector, such as the fact that patients often do not have opportunities to make a choice or that they are content with the default hospital. Therefore, policy makers may try in vain to bring principles originating from neoclassical microeconomic theory into health care. In health care, other principles seem to apply. The choice of a hospital is only one part of patients' health‐care paths. Other means to ensure that patients visit a hospital that matches their preferences should be devised. Perhaps, GPs might be able to acquire patients' preferences regarding a hospital and use these to, together with the patient, choose a hospital that fits these preferences.

## Funding

The Ministry of Education, Culture and Science of the Dutch government provided financial support for this research, but was not involved in any part of it.

## Conflict of interest

No conflict of interest.
